# Comprehensive Analysis Reveals Epithelial Growth Factor Receptor as a Potential Diagnostic Biomarker in Glioblastoma Multiforme

**DOI:** 10.7759/cureus.64506

**Published:** 2024-07-14

**Authors:** Amna Makawi, Somia A Khalafallah, Israa M Faris, Mohamed Alfaki

**Affiliations:** 1 Medicine, Elrazi University, Doha, QAT; 2 Hematology and Immunohematology, Ibn Sina University, Khartoum, SDN; 3 Fertilization and Artificial Insemination, Istanbul University-Cerrahpasa, Istanbul, TUR; 4 Research, Sidra Medicine, Doha, QAT

**Keywords:** biomarkers, genetics, bioinformatics, cancer, egfr, glioblastoma multiforme

## Abstract

Glioblastoma multiforme (GBM), a highly aggressive tumor of the central nervous system, is the most common malignant brain tumor and poses a significant risk to life. GBM patients have a low survival rate owing to their aggressive nature, poor prognosis, genomic variations among patients, and histopathological differences. In this study, we used several bioinformatics platforms, namely Tumor Immune Estimation Resource (TIMER), Gene Expression Profiling Interactive Analysis (GEPIA), University of Alabama at Birmingham Cancer Data Analysis Portal (UALCAN) databases, Kaplan-Meier plotter, and cBioPortal, to conduct a comprehensive analysis to highlight the expression of epithelial growth factor receptor (EGFR) in patients with GBM. Our study highlights EGFR as a potential diagnostic and prognostic marker. According to the TIMER database, EGFR was upregulated in five cancers, including GBM, head and neck squamous cell carcinoma, kidney renal cell carcinoma, kidney renal cell papillary cell carcinoma, and lung squamous cell carcinoma, whereas it was downregulated in breast invasive carcinoma, colon adenocarcinoma, pheochromocytoma and paraganglioma, prostate adenocarcinoma, rectum adenocarcinoma, and uterine corpus endometrial carcinoma. Our investigation highlighted the expression of EGFR in various clinicopathological parameters, which include age, sex, gender, and TP53 mutation status in patients with GBM. We found that EGFR was upregulated in middle-aged and older adults compared to normal tissues, while it was not significantly downregulated in young adults and older adults. EGFR was upregulated in Caucasians compared to normal tissue, whereas it was downregulated in Asian and African American populations, but this was not statistically significant. In terms of gender, EGFR was upregulated in the male population compared to the female population. Furthermore, EGFR was upregulated in patients with TP53 mutations compared to normal tissues. We also examined the correlation between EGFR gene expression and immune cell infiltration in GBM patients and the impact of EGFR mutations on patient prognosis. Our results revealed a significant positive correlation between EGFR, B cells, and macrophages, but this was not significant for other cell types. This study signified that upregulation of EGFR was associated with a poor prognosis in patients with GBM validated by the GEPIA and UALCAN databases.

## Introduction

One of the most serious and life-threatening tumors of the central nervous system is glioblastoma multiforme (GBM) [1]. Brain cancers are classified as either gliomas, i.e., originating from glial cells, or non-glioma tumors [2]. GBM is classified as a WHO-grade IV glioma, and it is the most common malignant brain tumor with a five-year survival analysis of 7.2% [3]; it is characterized based on its aggressive behavior, poor prognosis, and poor survival rate [1].

GBM can be classified into two subtypes: primary, which occurs in the elderly without any known preexisting disease, and secondary, which usually occurs in the younger population and is characterized by phosphate and tension homolog mutations, loss of heterozygosity, and amplification of the epithelial growth factor receptor (EGFR). Primary GBM is usually asymptomatic before malignancy, and it comprises 90% of the total GBM cases worldwide [1], whereas secondary GBM accounts only for 5% of the GBM [1].

Local amplification, alteration of EGFR, and dislocation of the gene have been found to be the most common genetic aberrations in GBM, which account for 57% of the total cases [1]. EGFR is a transmembrane receptor tyrosine kinase that is critical for normal development and function. EGFR amplification and mutation reprogram cellular metabolism and broadly alter gene transcription to drive tumor formation and progression, rendering EGFR a compelling drug target [4]. In this study, our aim is to systematically analyze the expression status, prognostic value, and genetic alteration of EGFR in patients who have GBM.

## Materials and methods

Transcriptional expression analysis of genes

Tumor Immune Estimation Resource (TIMER) 2.0

The TIMER database is an ideal resource for systematically analyzing associations between gene expression and tumor features in TCGA [5]. It uses a complex algorithm to determine the number of immune cell types based on expression patterns, enabling the study of immune infiltrates across various cancer types [6]. The transcriptional profiling of EGFR was compared between normal tissue and cancer tissue. We particularly highlight the association between GBM and EGFR. No normal tissue expression was mentioned. However, it is highly expressed in the cells of patients affected by GBM.

Gene Expression Profiling Interactive Analysis (GEPIA)

GEPIA provides an online tool for analyzing RNA sequencing expression data from the TCGA and the GTex studies. It has customizable features for differential expression analysis, profiling, graphing, and correlation analysis [7]. In this study, we used the GEPIA database to examine the EGFR expression in GBM to confirm the TIMER results. GEPIA was also used as a prognostic value for EGFR expression in GBM by plotting the Kaplan-Meier plots, which provided further information about its possible influence on patients’ outcomes.

University of Alabama at Birmingham Cancer Data Analysis (UALCAN) Portal

The UALCAN database is a user-friendly digital resource for analyzing cancer omics data. It analyzes biomarker discovery and gene validation, provides graphs and plots for gene expression and patient survival, and assesses epigenetic regulation [8]. In this study, we used the UALCAN database to examine the expression of EGFR in GBM cells to validate the expression in the GEPIA and TIMER databases. We also used this database to identify connections between EGFR and clinicopathological conditions like age, gender, sex, and race.

Kaplan-Meier Plotter

The Kaplan-Meier plotter is capable of assessing the correlation between the expression of all genes (mRNA, miRNA, protein, and DNA) and survival in 35,000 samples from 21 tumor types [9]. We used the Kaplan-Meier plotter to determine the survival analysis between EGFR and GBM.

cBioPortal

The cBioPortal for Cancer Genomics is a useful resource for interactively exploring genetic alterations and multidimensional cancer genomics data sets [10]. In this work, we used cBioPortal to look into the genetic changes of BMX across diverse cancer types.

Validation of EGFR expression using ggplot

We used public datasets from the National Center for Biotechnology Information. Using the GEO2R tool (https://www.ncbi.nlm.nih.gov/geo/geo2r), differential analysis was done. Tools are provided to help users query and download experiments and curated gene expression profiles [11]. This enabled us to identify the significance of EGFR in GBM by using the (GSE137900) dataset. We used ggplot2 to visualize the result using a volcano plot. ggplot2 is an R package for producing visualizations of data [12]. We have applied the criteria of log 2 fold change |Log2FC| > 1 and adj p-value < 0.05 to statistically analyze differentially expressed genes.

## Results

Pan-cancer analysis comparing the expression of EGFR in normal and tumor cells

**Figure 1:**
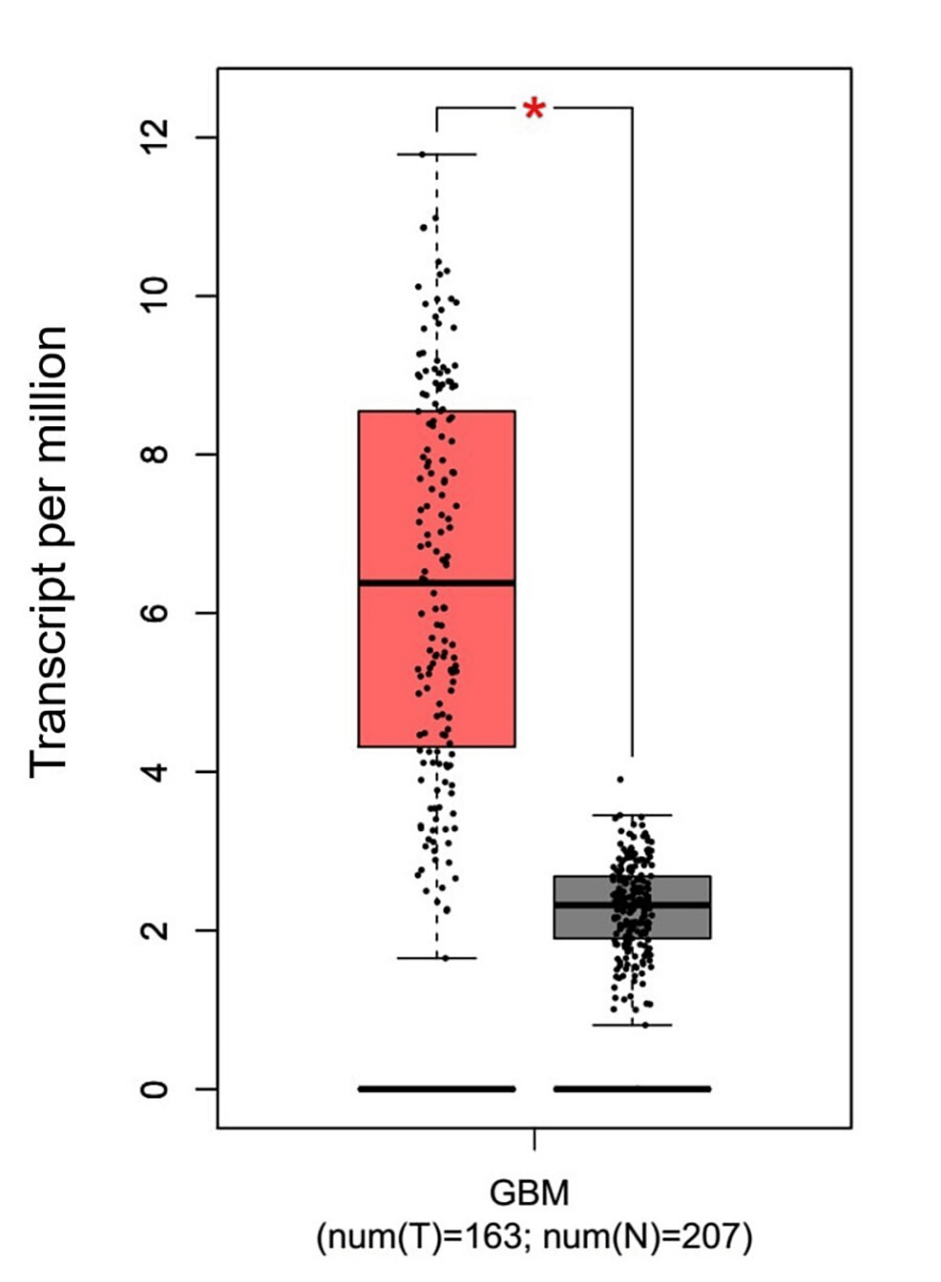
Expression of EGFR in GBM using the GEPIA database The red box indicates the expression of EGFR in GBM patients, while the gray box indicates the expression of EGFR in normal patients. num(T): number of tumor samples; num(N): number of normal samples *p < 0.05, **p < 0.01, ***p < 0.001 EGFR, epithelial growth factor receptor; GBM, glioblastoma multiforme; GEPIA, Gene Expression Profiling Interactive Analysis

**Figure 2:**
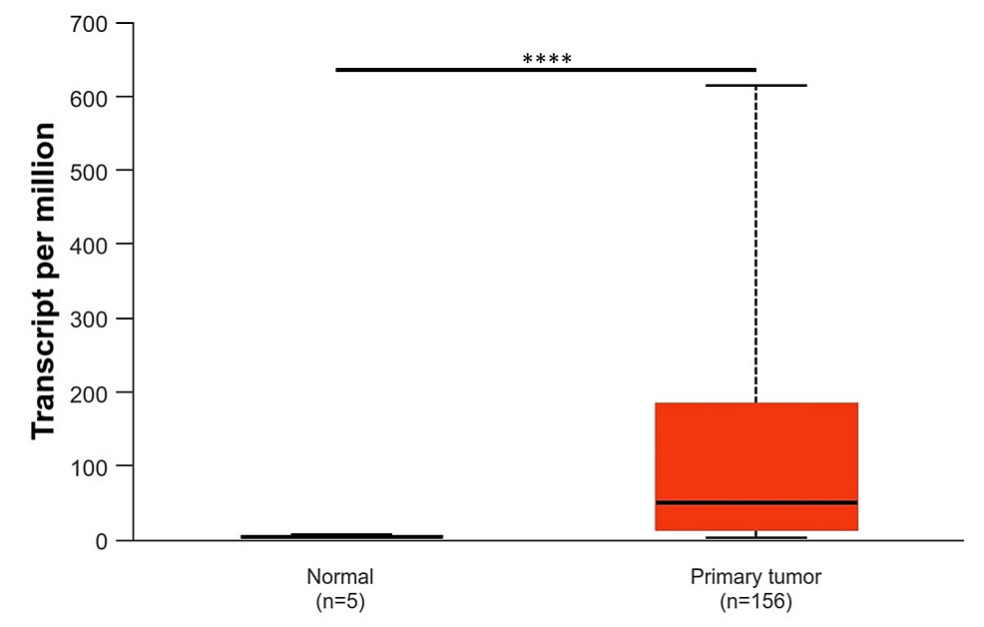
The UALCAN database shows increased expression of EGFR in primary tumors *p < 0.05, **p < 0.01, ***p < 0.001 EGGR, epithelial growth factor receptor; UALCAN, University of Alabama at Birmingham Cancer Data Analysis Portal

In this study, we conducted a comprehensive analysis of EGFR across GBM using three databases: TIMER, GEPIA, and UALCAN. This analysis revealed that the expression of EGFR was significant and highly expressed in the TIMER database (p = <0.01, num(N) = 5, and num(T) = 153). This was also the case in GEPIA (Figure 1) and UALCAN (Figure 2), with a p-value of <0.05, num(N) = 207, num(T) = 163, and <0.001, num(N) = 5, num(T) = 156, respectively.

**Figure 3:**
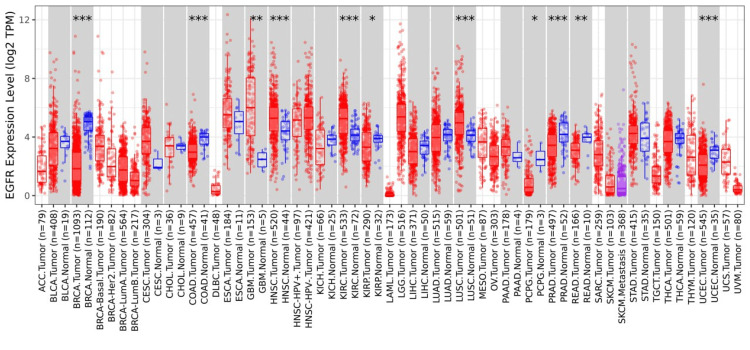
Pan-cancer analysis conducted to evaluate the expression of EGFR across various cancers EGFR expression using TIMER database *p < 0.05, **p < 0.01, ***p < 0.001 EGGR, epithelial growth factor receptor; TIMER, Tumor Immune Estimation Resource

We also analyzed the expression of EGFR across other cancers using the TIMER database (Figure 3), and the analysis revealed that this gene was statistically significant in 11 cancers, namely breast invasive carcinoma (BRCA), colon adenocarcinoma (COAD), GBM, head and neck squamous cell carcinoma (HNSC), kidney renal cell carcinoma (KIRC), kidney renal cell papillary cell carcinoma (KIRP), lung squamous cell carcinoma (LUSC), pheochromocytoma and paraganglioma (PCPG), prostate adenocarcinoma (PRAD), rectum adenocarcinoma (READ), and uterine corpus endometrial carcinoma (UCEC). Out of these, five of them were upregulated, namely, GBM, HNSC, KIRC, KIRP, and LUSC, and six were downregulated, namely, BRCA, COAD, PCPG, PRAD, READ, and UCEC.

Clinical parameter analysis of the EGFR gene across GBM

**Figure 4:**
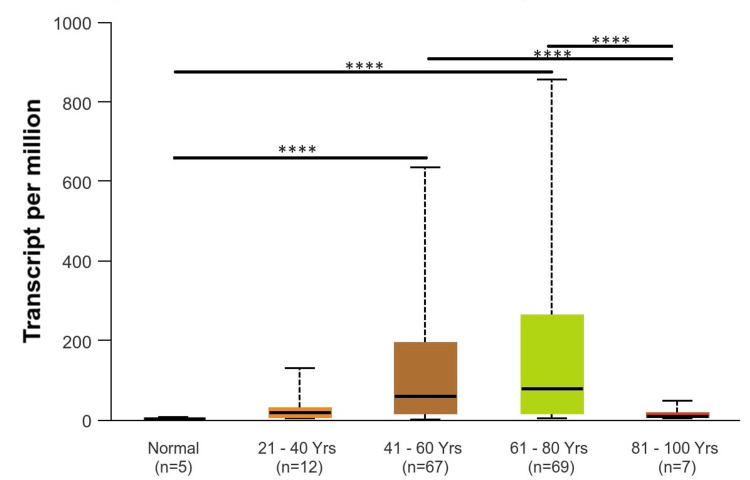
Representation of EGFR expression based on patient age *p < 0.05, **p < 0.01, ***p < 0.001 EGGR, epithelial growth factor receptor

The expression of EGFR and various clinicopathological parameters like age, race, gender, and TP-53 mutation status in GBM patients were analyzed. Figure 4 shows the expression of EGFR based on the patient’s age, where 21-40 years are classified as young adults, 41-60 years are classified as middle-aged adults, 61-80 years are classified as older adults, and 81-100 years are classified as elders. This figure shows that EGFR was upregulated and significant in middle-aged adults and older adults as compared to normal and as compared to elders, with a p-value of p < 0.001. EGFR was downregulated in young adults and elders with a p-value of >0.001, which is not statistically significant.

**Figure 5:**
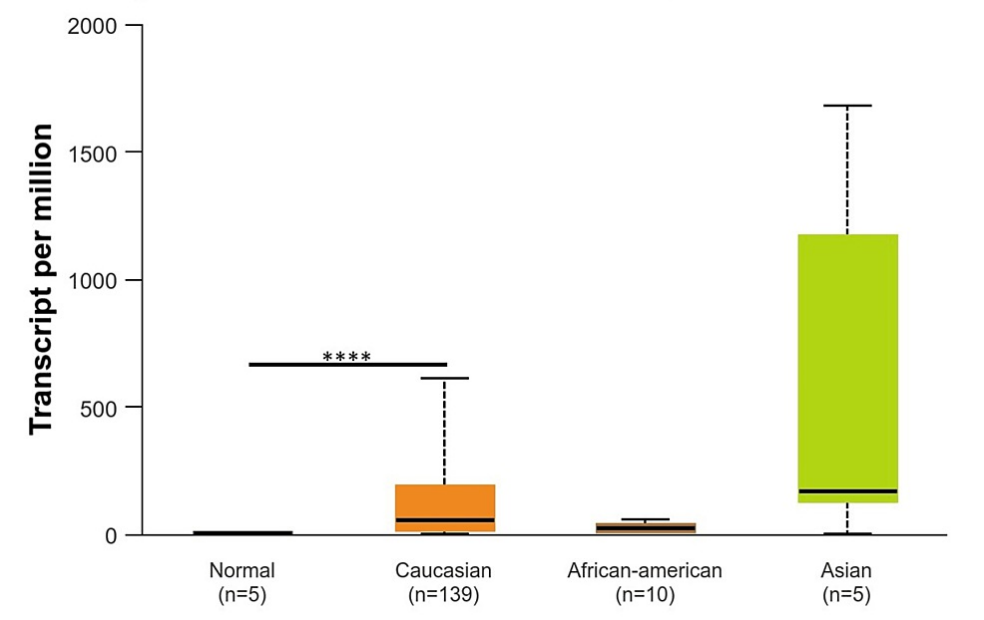
Representation of EGFR expression based on patient race *p < 0.05, **p < 0.01, ***p < 0.001 EGGR, epithelial growth factor receptor

**Figure 6:**
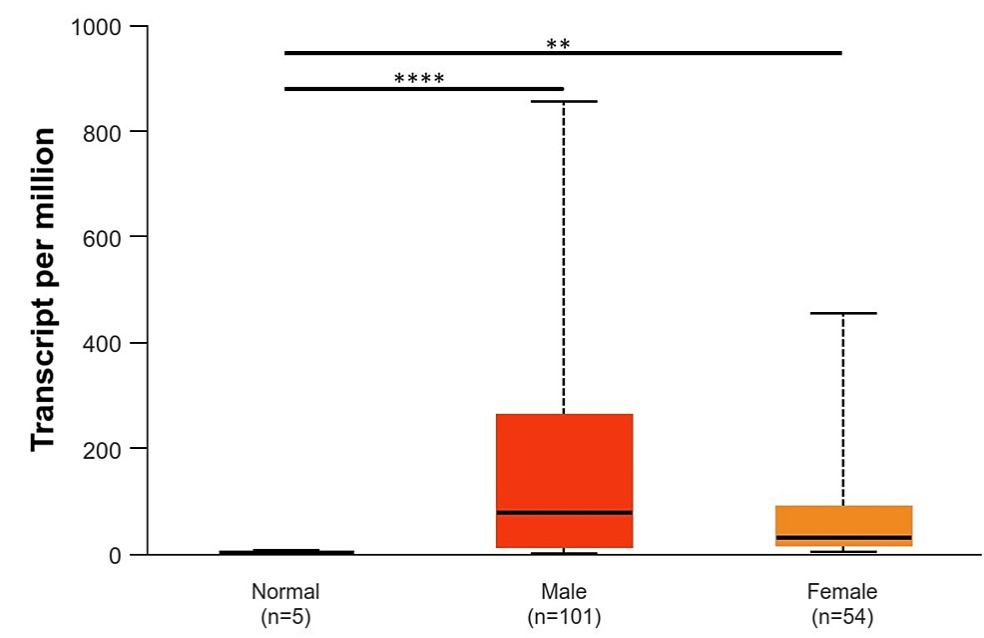
Representation of EGFR expression based on patient gender *p < 0.05, **p < 0.01, ***p < 0.001 EGGR, epithelial growth factor receptor

**Figure 7:**
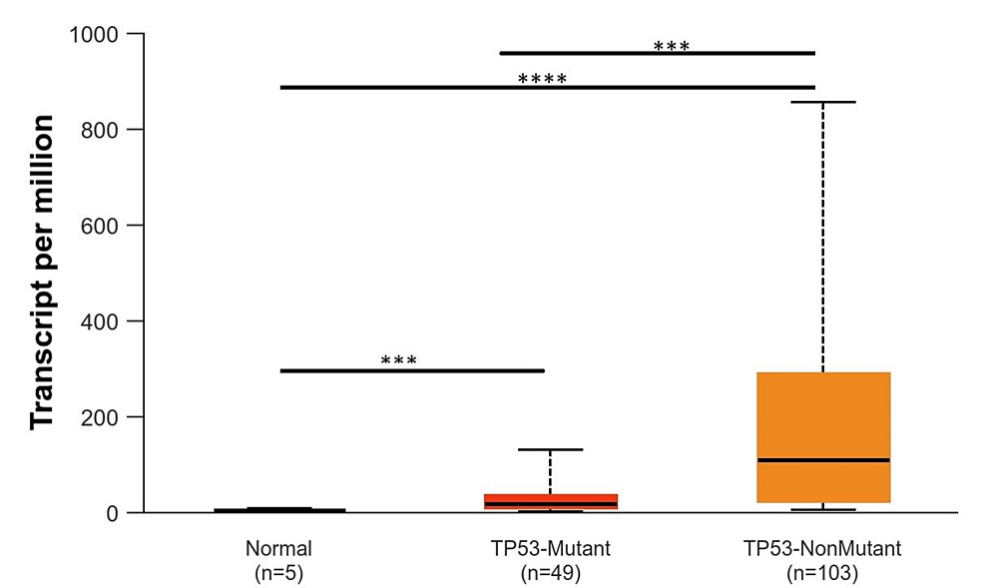
Representation of EGFR expression based on patients TP53-mutant status *p < 0.05, **p < 0.01, ***p < 0.001 EGGR, epithelial growth factor receptor

Based on patients’ races (Caucasians, African Americans, and Asians), it was found that EGFR was upregulated in Caucasians with a p-value of p < 0.001 as compared to normal tissue, while EGFR was downregulated in the Asian population and African American population with a p-value of p > 0.001, which was not significant (Figure 5). According to gender (male and female), EGFR was upregulated in the male population as compared to the female population. The p-value of the male population and female population as compared to normal was significant (p < 0.001) (Figure 6). According to TP53-mutation status, EGFR was statistically significantly upregulated in both patients who were TP53-mutant and non-mutant compared to the normal samples (p < 0.001). Also, there are statistically significant differences between patients who are TP53-mutant and non-mutant. The EGFR gene intends to be highly expressed in TP53 non-mutants more than in those who have a TP53 mutant in GBM (Figure 7).

EGFR is correlated with the abundance of immune cells

**Figure 8:**
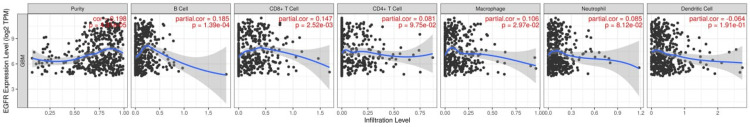
Correlation of EGFR expression among GBM tumors with immune cell filtration levels (macrophages, neutrophils, dendritic cells, B cells, CD8+ T cells, and CD4+ T cells) using TIMER database cor and p-value B cells, B lymphocytes; CD4+ T cells, T helper cells; CD8+ T cells, cytotoxic T lymphocytes; Cor, correlation; EGFR, epithelial growth factor receptor; GBM, glioblastoma multiforme; log2 TPM, log2 transcripts per million; partial cor, partial correlation; TIMER, Tumor Immune Estimation Resource

In this study, we used the TIMER database to correlate EGFR gene expression and immune cell infiltration. Several cells were included in this validation, namely B cells, CD8+ T cells, CD4+ T cells, macrophages, neutrophils, and dendritic cells, and tumor purity was also included in patients with GBM. This analysis showed that there is a statistically significant positive correlation in B cells (Cor = 0.185, p = 0.000139), CD8+ cells (Cor = 0.147, p = 0.0025), and macrophage cells (Cor = 0.106, p = 0.0297), while the other cells were not significant (p > 0.05) (Figure 8).

Representation of overall survival rates in patients with GBM

**Figure 9:**
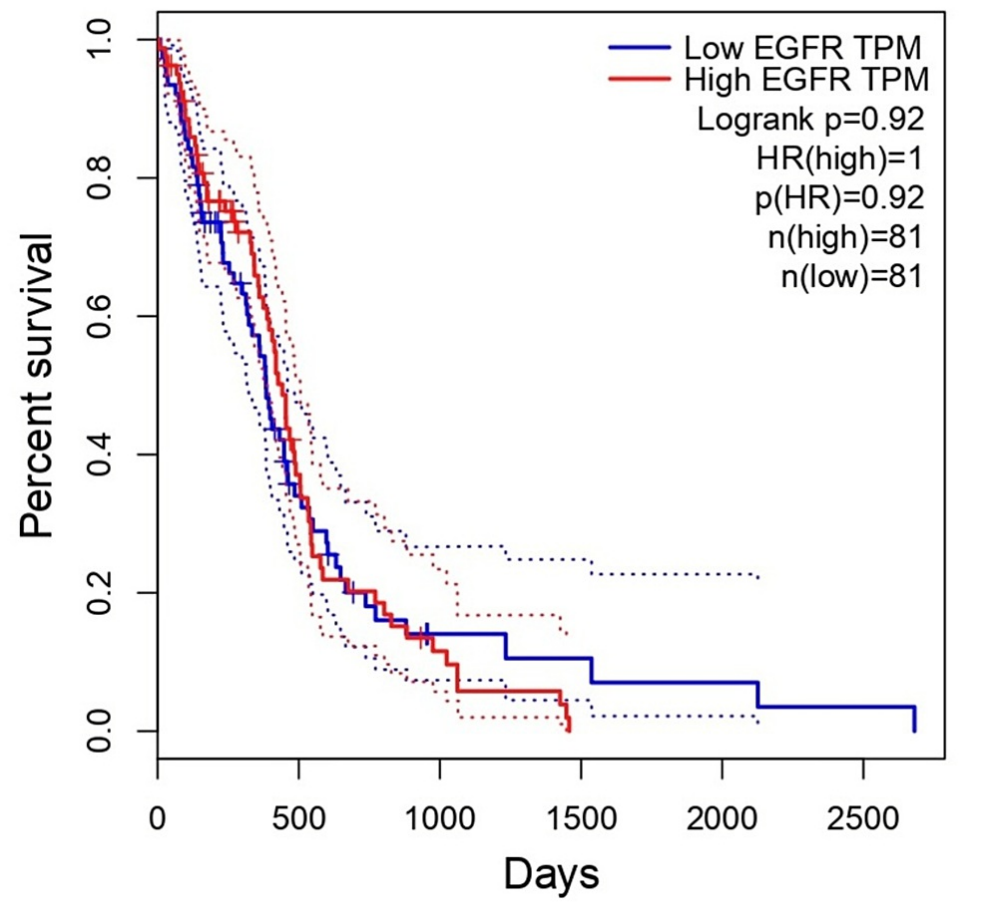
The correlation between the EGFR expression level and the survival outcome of patients using the GEPIA database EGFR, epithelial growth factor receptor; GBM, glioblastoma multiforme; GEPIA, Gene Expression Profiling Interactive Analysis; Log-rank P, p-value resulting from log-rank test; OS, overall survival; TPM, transcripts per million

**Figure 10:**
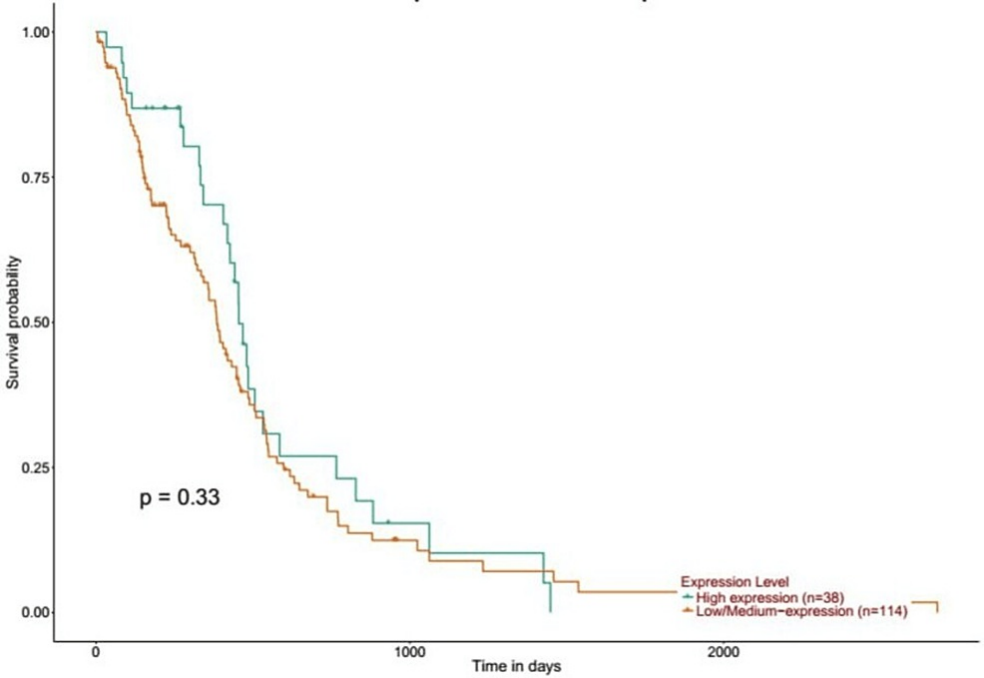
The correlation between the EGFR expression level and the survival outcomes of patients EGFR, epithelial growth factor receptor; GBM, glioblastoma multiforme; UALCAN, University of Alabama at Birmingham Cancer Data Analysis Portal

The correlation between EGFR expression and patients affected by GBM was explored using GEPIA (Figure 9) and UALCAN (Figure 10). The below figure shows that upregulation of the EGFR gene was associated with a poor prognosis in GBM-affected patients: HR = 1, p(HR) = 0.9, n (high) = 81, n (low) = 81 (Figure 9); UALCAN; p = 0.33, high expression (n) = 38, low/medium expression (n) = 114 (Figure 10).

Genetic variation of EGFR across various cancers

**Figure 11:**
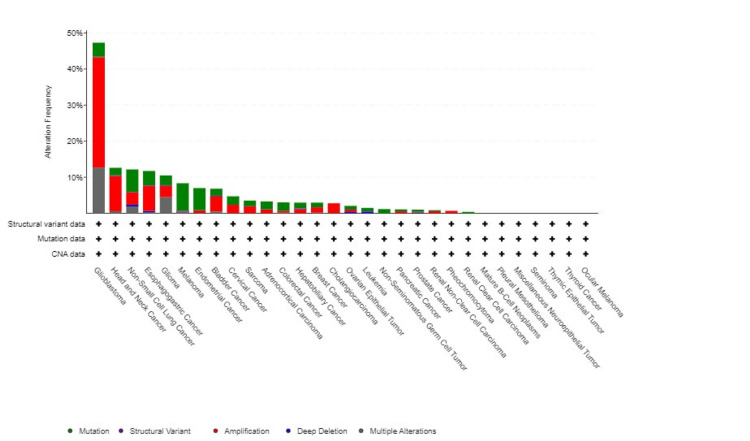
Genetic alteration analysis of EGFR using the cBioPortal database EGGR, epithelial growth factor receptor

**Figure 12:**
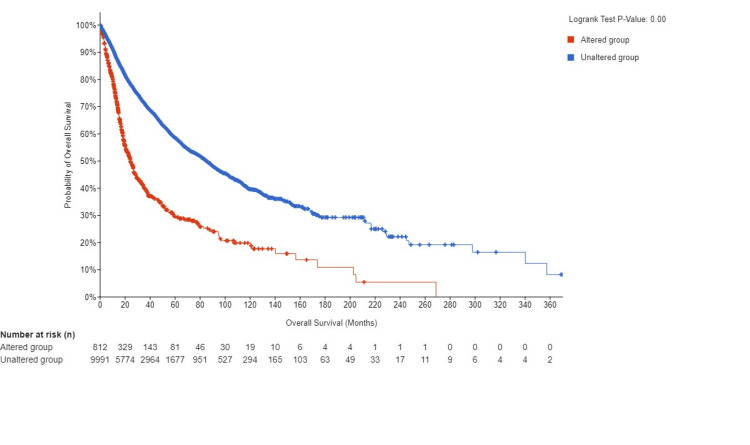
Genetic alteration analysis of EGFR using the cBioPortal database EGGR, epithelial growth factor receptor; log-rank P: p-value resulting from log-rank test

Using the cBioPortal platform, the genetic variation of EGFR across various cancers was analyzed. We utilized the TCGA database to determine this expression. We have found that EGFR was mutated in 7% of the samples that we queried; this included 10,967 samples from 32 different studies. Our findings showed that EGFR was mostly amplification mutations and multiple mutations. We also found that most EGFR mutations occurred in GBM (amplification frequency = 30.57% (181 cases), multiple alteration = 12.67% (75 cases), mutation = 3.89 (23 cases), and structural variant = 0.17 (one case)) (Figure 11). We have found that EGFR was not mutated in some samples; however, patients with mutated EGFR had a worse prognosis than those who did not have a mutated EGFR with a significant p-value (p = 0.00) (Figure 12).

Cross-validation of EGFR using ggplot

**Figure 13:**
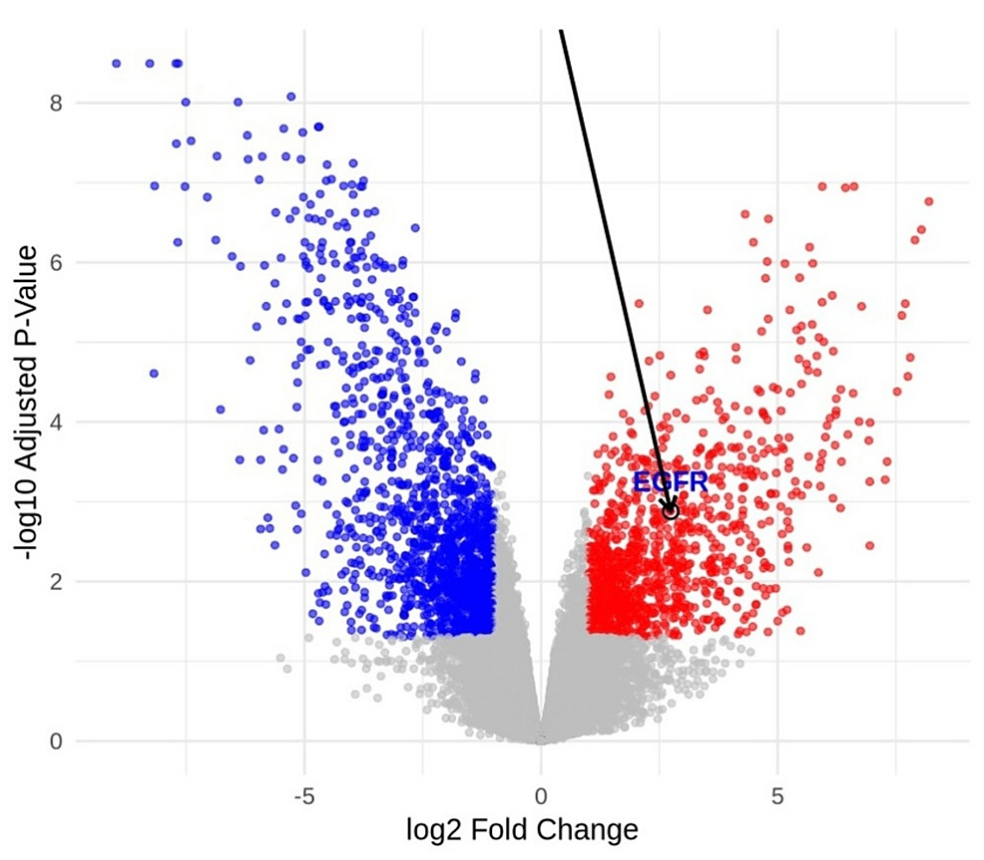
Volcano plots illustrate the differentially expressed genes and annotation of EGFR The red dots indicate upregulated genes, the blue dots indicate downregulated genes, and the gray dots indicate nonsignificant genes. EGFR, epithelial growth factor receptor

Using public databases that were obtained from Gene Expression Omnibus (GEO), we validated the expression of EGFR in GBM patients. The GEO2R tool was used to obtain the expression of EGFR (|Log2FC| > 1 and adjusted p-value < 0.05). We then utilized the R analysis to visualize the volcano plot for differentially expressed genes. The database used included three normal brain tissues and six brain tissues affected by GBM. The analysis included a total of 2,666 genes, out of which 1,136 were upregulated and 1,530 were downregulated. Figure 13 shows the upregulation of EGFR in GBM patients using the volcano plot. We found that the EGFR was significantly upregulated (log2FC = 2.7, adj p-value = 0.001).

## Discussion

The aim of this study was to investigate EGFR as a potential diagnostic biomarker for patients with GBM using TIMER, GEPIA, and UALCAN. As therapeutic decisions are increasingly guided by biomarkers and EGFR abnormalities are common in GBM, we wanted to study the expression of the gene to highlight its importance as a potential therapeutic target.

In this study, we have observed that the expression of EGFR between normal and tumor samples was upregulated and statistically significant in all three databases above. Furthermore, UALCAN was used to establish the association between the expression of EGFR and different clinicopathological parameters like age, race, gender, and TP53 status in GBM-affected patients.

Our study shows that the level of EGFR in normal samples was significantly upregulated in middle-aged adults and older adults as compared to normal. In terms of race, it was found that EGFR was highly expressed only in the Caucasian population compared to the normal samples. According to gender, males and females both showed highly significant upregulation of the EGFR compared to normal samples. In TP53-mutation status, expression of EGFR was found to be highly upregulated in patients who were TP53 non-mutant as well as TP53-mutant patients compared to normal. The study also suggests that in GBM, patients with (wild-type) TP53 non-mutated have a statistically significant increase in EGFR upregulation compared to those with TP53-mutant, although the impact of the cancer stage on this association remains unclear.

This study identified significant positive correlations between EGFR expression and various B cell, CD8+, and macrophage immune cell infiltrates, suggesting their potential as prognostic and therapeutic biomarkers. While no statistical correlations were found between EGFR expression and GBM patient outcomes using GEPIA and UALCAN, high EGFR expression was still associated with a poor prognosis in GBM patients. A previous study showed that EGFR-mutant cases exhibit increased infiltration of CD4+ T cells, neutrophils, macrophages, and dendritic cells, leading to a poor prognosis in low-grade gliomas [13]. Increased infiltration of these immune cells in EGFR-mutant cases was significantly correlated with shorter survival times [13].

Another study done by Xu et al. showed that EGFR family members, including EGFR and ERBB2, exhibit higher mRNA expression levels in GBM. In addition, higher mRNA levels of EGFR and ERBB2 were linked to increased immune infiltration in glioblastoma [14], suggesting their potential as therapeutic targets and prognostic markers in GBM. Understanding EGFR expression and its related pathways is crucial for developing effective targeted therapies and improving outcomes for GBM patients. Our study supports the idea that EGFR expression is intricately linked to TP53 mutation status in GBM. The observed finding that patients with non-mutated TP53 (wt-p53) exhibit statistically significant upregulation of EGFR aligns with previous research demonstrating that EGFR signaling can inhibit wt-p53 function, which was done by Ding et al. [15]. Further investigation into the underlying mechanisms and potential therapeutic implications of this interplay between EGFR and TP53 is warranted.

We have also studied the overall survival of EGFR in patients with GBM using the GEPIA and UALCAN databases, and it showed that patients with high expression of EGFR were associated with a poor prognosis. This result was supported by a study that aimed to analyze the expression of EGFL17 (epithelial growth factor like multiple 7) in GBM using immunohistochemistry and in silico methods, which also showed that high EGFR expression was associated with poor overall survival in GBM patients, emphasizing its potential as a prognostic indicator [16,17].

We used the cBioPortal platform to study the mutation of EGFR in patients with GBM, and we found that most of the mutation was an amplification mutation (30.5%), followed by multiple alterations (12.67%). This highlighted the fact that patients with non-mutant EGFR genes had better prognoses than patients with mutant EGFR genes. To cross-validate our results, we have used a ggplot using R analysis to establish a volcano plot to highlight the upregulation of this gene in patients with GBM.

In this study, we integrated three normal brain samples and six brain samples affected by GBM to cross-validate our results using ggplot, and we found that EGFR was upregulated in patients with GBM.

Limitations of the study

While using EGFR copy number and expression data from The Cancer Genome Atlas (TCGA) offers valuable insights into its potential role as a biomarker in glioblastoma (GBM), there are important limitations to consider. TCGA data represents a specific patient population, potentially excluding subgroups with unique EGFR profiles or clinical characteristics. In addition, GBM is a highly heterogeneous tumor type with varying genetic and molecular profiles. Focusing solely on EGFR might overlook other relevant biomarkers that could be crucial for patient stratification. This may limit the generalizability of the findings to the entire GBM population.

Despite these limitations, using TCGA data provides a valuable starting point for understanding the potential of EGFR as a biomarker in GBM. Future studies that address these limitations, incorporating prospective clinical trials and focusing on specific patient subgroups, are crucial for validating the clinical utility of EGFR as a biomarker for GBM diagnosis, prognosis, and treatment stratification.

## Conclusions

The mutation of the EGFR gene is more indicative of a poor prognosis than its upregulation. Additionally, patients with EGFR mutations have a more unfavorable prognosis than those with elevated EGFR expression. In this study, we aimed to provide the prognostic value of EGFR in patients affected by GBM as a possible therapeutic target to treat this aggressive and fatal malignancy. However, wet lab experiments with GBM-affected cells are required to highlight this conclusion.

## References

[1] Jadoon SS, Ilyas U, Zafar H (2022). Genomic and epigenomic features of glioblastoma multiforme and its biomarkers. J Oncol.

[2] Mahmoud AB, Ajina R, Aref S (2022). Advances in immunotherapy for glioblastoma multiforme. Front Immunol.

[3] Wu W, Klockow JL, Zhang M (2021). Glioblastoma multiforme (GBM): an overview of current therapies and mechanisms of resistance. Pharmacol Res.

[4] Liu F, Mischel PS (2018). Targeting epidermal growth factor receptor co-dependent signaling pathways in glioblastoma. Wiley Interdiscip Rev Syst Biol Med.

[5] Li B, Severson E, Pignon JC (2016). Comprehensive analyses of tumor immunity: implications for cancer immunotherapy. Genome Biol.

[6] Li T, Fan J, Wang B (2017). TIMER: a web server for comprehensive analysis of tumor-infiltrating immune cells. Cancer Res.

[7] Tang Z, Li C, Kang B, Gao G, Li C, Zhang Z (2017). GEPIA: a web server for cancer and normal gene expression profiling and interactive analyses. Nucleic Acids Res.

[8] Chandrashekar DS, Bashel B, Balasubramanya SA, Creighton CJ, Ponce-Rodriguez I, Chakravarthi BV, Varambally S (2017). UALCAN: a portal for facilitating tumor subgroup gene expression and survival analyses. Neoplasia.

[9] Győrffy B (2024). Transcriptome-level discovery of survival-associated biomarkers and therapy targets in non-small-cell lung cancer. Br J Pharmacol.

[10] de Bruijn I, Kundra R, Mastrogiacomo B (2023). Analysis and visualization of longitudinal genomic and clinical data from the AACR Project GENIE Biopharma Collaborative in cBioPortal. Cancer Res.

[11] Edgar R, Domrachev M, Lash AE (2002). Gene Expression Omnibus: NCBI gene expression and hybridization array data repository. Nucleic Acids Res.

[12] Villanueva RA, Chen ZJ (2019). ggplot2: elegant graphics for data analysis. Measurement (Mahwah N J).

[13] Hao Z, Guo D (2019). EGFR mutation: novel prognostic factor associated with immune infiltration in lower-grade glioma; an exploratory study. BMC Cancer.

[14] Xu B, Huo Z, Huang H (2021). The expression and prognostic value of the epidermal growth factor receptor family in glioma. BMC Cancer.

[15] Ding J, Li X, Khan S (2022). EGFR suppresses p53 function by promoting p53 binding to DNA-PKcs: a noncanonical regulatory axis between EGFR and wild-type p53 in glioblastoma. Neuro Oncol.

[16] da Costa BH, Becker AP, Neder L (2022). EGFL7 expression profile in IDH-wildtype glioblastomas is associated with poor patient outcome. J Pathol Transl Med.

[17] Shi Y, Sun Y, Cheng H, Wang C (2021). EFNB1 acts as a novel prognosis marker in glioblastoma through bioinformatics methods and experimental validation. J Oncol.

